# SARS-CoV-2-Induced IgG Repertoires Shape Gamma-Delta T Cell Responses: Evidence for Direct IgG-Membrane Interaction According to Disease Severity

**DOI:** 10.3390/cells15050401

**Published:** 2026-02-26

**Authors:** Anna Luisa Baratelli Moreira, Nicolle Rakanidis Machado, João Vitor da Silva Borges, Lais Alves do Nascimento, Beatriz Oliveira Fagundes, Nátali Espasiani Cilento, Carolina Nunes França, Maria Notomi Sato, Marilia Garcia de Oliveira, Jefferson Russo Victor

**Affiliations:** 1 Laboratory of Medical Investigation LIM-56, Division of Dermatology, Medical School, University of São Paulo, São Paulo 05403-000, Brazil; annaluisabm21@gmail.com (A.L.B.M.); laisalves01711@gmail.com (L.A.d.N.); b.oliveira.fagundes@umcg.nl (B.O.F.); marisato@usp.br (M.N.S.); 2 Medical School, Santo Amaro University (UNISA), São Paulo 04829-300, Brazil; joaovitor953borges@gmail.com; 3 Department of Dermatology, University Medical Center Groningen, 9713 GZ Groningen, The Netherlands; 4 Post Graduation Program in Health Sciences, Santo Amaro University (UNISA), São Paulo 04829-300, Brazilcnfranca@prof.unisa.br (C.N.F.); 5 Ann Romney Center for Neurologic Diseases, Brigham and Women’s Hospital, Harvard Medical School, Boston, MA 02115, USA

**Keywords:** SARS-CoV-2, COVID-19, IgG repertoire, γδ T cells, cytokines, autoreactivity, immune modulation, disease severity

## Abstract

**Highlights:**

**What are the main findings?**

- SARS-CoV-2 infection generates IgG repertoires that directly interact with γδ T cells, reshaping their phenotype, homing profile, and cytokine responses in a disease severity-dependent manner.
- Severe COVID-19 is associated with expanded IgG idiotype networks that recognize γδ T cell-expressed proteins and map to inflammatory and immune-regulatory signaling pathways.

**What are the implications of the main findings?**

- These data position γδ T cells as interpreters of antibody-encoded immune information, revealing a previously underappreciated layer of humoral–unconventional T cell crosstalk in COVID-19.
- Profiling IgG idiotype repertoires may open new conceptual avenues for understanding and modulating immune dysregulation in viral infections beyond antigen-centric paradigms.

**Abstract:**

Immunoglobulin G (IgG) is a central component of humoral immunity in coronavirus disease 2019 (COVID-19); however, increasing evidence suggests that infection-induced IgG repertoires exert immunomodulatory effects beyond classical antiviral functions. In this study, we investigated whether IgG from patients with moderate or severe COVID-19 directly modulates human peripheral γδ T cells and whether these effects are associated with disease severity-dependent IgG idiotype profiles. Purified IgG from non-exposed healthy controls, moderate COVID-19 patients, or severe COVID-19 patients was incubat-ed with peripheral blood mononuclear cells from healthy donors. γδ T cell phenotype, subset distribution, homing markers, and cytokine production were assessed by flow cytometry, while direct IgG–cell interactions were evaluated using fluorescent IgG binding assays. In parallel, proteomic profiling using human proteome microarrays was performed to identify γδ T cell-expressed protein targets recognized by COVID-19-induced IgG. IgG from SARS-CoV-2-infected individuals selectively reduced Vγ9^+^Vδ2^+^ γδ T cells, altered memory differentiation, downregulated CCR5, CCR6, and CD161 expression, and reshaped cytokine production in a severity-dependent manner. COVID-19 IgG bound directly to the γδ T cell membrane without inducing apoptosis, indicating a non-cytotoxic mechanism of modulation. Proteomic analysis revealed a marked expansion and diversification of γδ T cell-associated IgG targets in COVID-19, particularly in severe disease, with enrichment of pathways related to immune signaling and inflammation. Collectively, these findings identify γδ T cells as direct functional targets of SARS-CoV-2-induced IgG repertoires and demonstrate that disease severity shapes IgG idiotype networks with distinct immunomodulatory capacities. This work highlights a previously underappreciated antibody-mediated mechanism contributing to immune dysregulation in COVID-19.

## 1. Introduction

Since the emergence of the COVID-19 pandemic [1], caused by severe acute respiratory syndrome coronavirus 2 (SARS-CoV-2), the disease has resulted in millions of deaths worldwide, according to estimates from international public health authorities. Over the past several years, substantial progress has been made in elucidating various aspects of SARS-CoV-2 infection, including its epidemiology, pathogenesis, and epigenetic influences, as well as developments in emergency clinical management, diagnostic approaches, vaccination strategies, and experimental models [2,3,4,5]. The humoral immune response to SARS-CoV-2 is primarily mediated by IgG, the predominant immunoglobulin isotype in human peripheral blood. IgG is known to modulate the activity of diverse immune cell types, including T and B lymphocytes, across several pathological contexts such as allergic disorders [6,7], dermatological diseases [8], and viral infections [9]. In COVID-19, emerging evidence suggests that anti-SARS-CoV-2 IgG can influence the function of mucosal-associated invariant T (MAIT) cells in vitro, supporting the hypothesis that infection-induced antibodies may contribute to disease manifestations and post-acute COVID-19 complications [10,11].

A recent proteome-wide study using a human proteome microarray—encompassing more than 21,000 unique human proteins derived from 16,794 genes (representing over 81% of the annotated human proteome)—identified a complex network of SARS-CoV-2-induced IgG antibodies with distinct antigenic specificities, hereafter referred to as idiotypes. Notably, these idiotypes recognized a broad range of human proteins beyond what would be predicted by simple cross-reactivity with homologous sequences, indicating previously unanticipated interactions between infection-induced antibodies and the host proteome [12]. Another study further suggested that SARS-CoV-2-induced IgG idiotypes may interact with proteins expressed across nearly all peripheral lymphoid and myeloid cell populations, although it did not evaluate direct IgG–membrane engagement or the potential functional consequences of such interactions [13]. Additionally, recent findings indicate that the IgG idiotype repertoire elicited in patients with moderate or severe COVID-19 not only differs in its recognition of SARS-CoV-2 epitopes but also exhibits distinct reactivity patterns toward dozens of other pathogen-derived epitopes, including those from viruses, bacteria, parasites, and fungi [14].

γδ T lymphocytes constitute a distinct subset of T cells characterized by their expression of γδ T cell receptors (γδTCRs). They represent the smallest circulating T cell population, accounting for approximately 0.5–5% of peripheral blood T cells [15]. Upon activation, γδ T cells can produce a broad repertoire of cytokines associated with classical T-helper lineages—including IFN-γ (Th1), IL-4 and IL-10 (Th2), IL-17 (Th17), and IL-22 (Th22)—thereby exerting wide-ranging immunomodulatory effects across innate and adaptive immunity [16,17,18,19,20,21].

Human γδ T cells are classically classified based on δ-chain usage into Vδ1^+^, Vδ2^+^, and Vδ3^+^ subsets. Vγ9^+^Vδ2^+^ cells predominate in peripheral blood, whereas tissue-resident subsets—particularly Vδ1^+^ cells—are enriched in mucosal and epithelial compartments [22,23,24]. γδ T cells are capable of adopting both type 1 and type 2 effector programs, and their immunological functions may vary substantially, sometimes even exhibiting opposing roles, depending on the subset identity and the surrounding cytokine milieu [25,26,27]. Developmentally, Vδ1^+^ γδ T cells are abundant in the fetal circulation and umbilical cord blood, but after birth the γδ T cell compartment undergoes a major shift, with progressive reduction in Vδ1^+^ cells and expansion of Vγ9^+^Vδ2^+^ cells, which ultimately comprise more than 75% of γδ T cells in adult peripheral blood [28].

Several studies have reported that γδ T cell frequencies in the peripheral blood of hospitalized COVID-19 patients are markedly reduced compared with healthy controls [29,30,31], identifying γδ T cells as one of the most profoundly affected lymphocyte subsets during SARS-CoV-2 infection [32]. In addition to this numerical decline, hospitalized patients exhibit a pronounced shift toward effector memory γδ T cell phenotypes at the time of admission, in contrast to the distributions observed in healthy donors [29,33,34]. The enrichment of memory-like γδ T cells suggests active recruitment of effector γδ T cells from the circulation to infected tissues—particularly the lungs—further supporting their involvement in the immune response to SARS-CoV-2 [35]. These observations underscore the need for mechanistic studies to disentangle the direct effects of SARS-CoV-2 infection on γδ T cell phenotype, distribution, and functional programming.

In parallel, growing evidence supports the concept that IgG antibodies may act as natural ligands for lymphocytes, modulating their activation state and functional properties according to the immunological background of the IgG donor [36]. Despite these advances, the molecular mechanisms and specific protein targets through which IgG interacts with and modulates T and B lymphocytes remain insufficiently defined. Nonetheless, the cumulative evidence indicates that T cells, particularly γδ T cells, may represent direct and functionally relevant targets of IgG-mediated modulation. Collectively, these findings highlight a broader, context-dependent role for IgG in shaping immune responses and pathogenic processes, extending beyond classical antigen specificity and occurring even in the absence of exogenous antigens.

Building on these observations, and with the aim of advancing immunomodulatory applications of IgG, we designed the present study to investigate the functional effects of IgG anti-SARS-CoV-2 from COVID 19 moderate or severe patients on peripheral γδ T cells from healthy donors. In parallel, we sought to identify the γδ T cell protein targets recognized by COVID-19-induced IgG using proteomic profiling, yielding unprecedented insights into IgG–γδ T cell interactions in the context of SARS-CoV-2 infection.

## 2. Methods

### 2.1. Sample Collection

Blood specimens were obtained from the Central Laboratory Division of the Clinical Hospital, Faculty of Medicine, University of São Paulo (São Paulo, Brazil). Serum was separated by centrifugation and stored at −20 °C until testing. Eligibility for inclusion required laboratory confirmation of SARS-CoV-2 infection by RT-PCR. Individuals older than 75 years or those lacking molecular confirmation were excluded.

The final cohort consisted of 79 patients with COVID-19 (39 men and 40 women). Disease severity was determined in accordance with the World Health Organization Clinical Management of COVID-19: Living Guideline (18 August 2023). Participants hospitalized with viral pneumonia who did not fulfill criteria for severe or critical disease—and who either required no oxygen therapy or received low-flow oxygen via nasal cannula or mask—were assigned to the moderate COVID-19 group (COVID-Mod; *n* = 39; mean age 41.6 ± 6.0 years; 17 men, 22 women). Patients who required high-flow oxygen support or non-invasive ventilation due to severe pneumonia were categorized as severe COVID-19 (COVID-Sev; *n* = 40; mean age 41.8 ± 6.0 years; 23 men, 17 women). Samples were collected between May and July 2020. Patients with critical manifestations of COVID-19 were not enrolled.

Control serum samples were obtained from 40 healthy volunteers (17 men and 23 women; mean age 28.5 ± 2.3 years) who donated blood prior to the COVID-19 pandemic (March–July 2019), designated as non-exposed healthy controls (Non-exp HCs). Additionally, peripheral blood was collected from 10 healthy donors (5 men and 5 women) between August and December 2022 for PBMC isolation. These donors had no prior COVID-19 diagnosis and were confirmed SARS-CoV-2 negative at the time of sampling. PBMCs were isolated on the same day using Ficoll-Paque density-gradient centrifugation (540× *g* for 20 min at 21 °C). Expanded demographic and clinical information is provided in Supplementary Table S1.

### 2.2. IgG Purification

Serum-derived immunoglobulin G (IgG) was isolated using the Melon Gel IgG Spin Purification Kit (Thermo Fisher Scientific, Waltham, MA, USA) in accordance with the manufacturer’s recommendations. Following purification, IgG preparations were passed through 0.20 µm sterile syringe filters (Corning, Darmstadt, Germany) and stored at −80 °C until use in downstream cell culture experiments. Protein concentrations were quantified using the Bradford-based Coomassie Protein Assay (Pierce, Thermo Fisher Scientific) following standard procedures. The purity of IgG preparations was evaluated by SDS–PAGE under reducing conditions and consistently exceeded 95%. Residual proteins, when present, were restricted to low-molecular-weight species (<10 kDa), indicating the absence of intact biologically active contaminants such as cytokines. The lack of IgA, IgM, and IgE contamination was confirmed by ELISA, and IgG subclass distributions were assessed and found to be comparable among all IgG pools. This purification approach efficiently enriched functionally intact IgG while minimizing immune complexes and aggregated immunoglobulin species. For biosafety purposes, all serum samples underwent ultraviolet irradiation and prolonged freezing prior to IgG purification to ensure inactivation of potential residual viral material associated with SARS-CoV-2 infection.

### 2.3. IgG Binding Assay and Assessment of γδT Cell Apoptosis

Purified IgG was fluorescently labeled using the Zenon™ Human IgG Labeling Kit (Invitrogen, Carlsbad, CA, USA), which employs monovalent Fab fragments directed to the IgG Fc region, minimizing unwanted interactions with other serum proteins. PBMCs were incubated for 15 min with 100 µg/mL of labeled IgG. Controls included cells exposed either to Zenon reagents without IgG or to unlabeled IgG alone.

Specificity of binding was verified by pre-incubating PBMCs with an excess of unlabeled IgG, which effectively prevented subsequent recognition by labeled IgG. The concentration used in functional assays was selected after preliminary titration experiments.

To assess plasma-membrane engagement and early apoptosis in γδ T cells, PBMCs were co-stained with Zenon-PE–labeled IgG, Annexin V–APC, Sytox Green, and antibodies against CD3–BV605 (clone UCHT1; Cat# 406-0038-41) and the γδ T cell receptor–PerCP-eFluor 710 (clone B1.1; Cat# 46-9959-41). This staining strategy enabled reliable discrimination between viable and apoptotic γδ T cell populations.

### 2.4. Cell Culture and Flow Cytometry

PBMC culture conditions followed previously established protocols [37]. Cells were cultured under one of the following conditions: no IgG (Mock), therapeutic polyvalent IgG (pIgG), IgG from Non-exp HCs, IgG from moderate COVID-19 patients, or IgG from severe COVID-19 patients. A total of 2 × 10^6^ PBMCs were plated per well in 48-well plates (Costar, Corning Life Sciences, Glandale, AR, USA) containing RPMI-1640 supplemented with 10% fetal bovine serum (HyClone III, Thermo Fisher Scientific, Logan, UT, USA) in a final volume of 400 µL. Cultures were maintained for 72 h with or without 100 µg/mL of the respective IgG preparation. pIgG was used as a reference control in selected functional assays. Intracellular cytokine staining protocol, encompassing defined time points, reagent concentrations, and detection antibodies, was previously standardized and recently published by our group [38].

γδ T cell phenotyping was performed by surface staining to identify total γδ T cells and specific subsets, including γδTCR–PerCP-eFluor 710 (clone B1.1; Cat# 46-9959-41), Vδ1–PE-Cy7 (clone TS8.2; Cat# 25-5679-42), Vδ2–BV421 (clone B6; Cat# 331428), and the Vγ9 chain–APC (clone B3; Cat# B381834). Memory differentiation was assessed based on the combined expression of CD45RA–NovaFluor Red 710 (clone HI100; Cat# H014T03R04-A) and CD27–BV605 (clone O323; Cat# 406-0279-42), allowing classification into naïve, central memory, effector memory, and terminally differentiated effector memory subsets. In addition, expression of CCR5–PE (clone NP-6G4; Cat# 12-1956-42), CCR6–PE-eFluor 610 (clone R6H1; Cat# 61-1969-42), and CD161–Alexa Fluor 488 (clone HP-3G10; Cat# 53-1619-42) was evaluated to assess homing potential and activation status.

For intracellular cytokine analysis, PBMCs were cultured under identical conditions, and Brefeldin A was added 6 h prior to harvest to inhibit cytokine secretion. After surface staining, cells were permeabilized with saponin and incubated with antibodies specific for IFN-γ–APC (clone 4S.B3; Cat# 551385), IL-4–PE (clone 8D4-8; Cat# 559333), IL-9–PerCP-Cy5.5 (clone MH9A3; Cat# 561461), IL-10–PE-Cy7 (clone JES3-9D7; Cat# 25-7108-42), IL-17–Alexa Fluor 700 (clone N49-653; Cat# 560613), and IL-22–PE-Cy7 (clone 22URTI; Cat# 25-7229-42). Cell viability was assessed using the PE–Texas Red Fixable Live/Dead Cell Stain (Thermo Fisher Scientific, Logan, UT, USA). All fluorochrome-conjugated antibodies were titrated in advance, and approximately 1 µg per test was determined to provide optimal resolution between positive and negative populations. Isotype controls and fluorescence-minus-one (FMO) controls were used to define gating boundaries.

Flow-cytometric acquisition was performed on an LSR Fortessa instrument (BD Biosciences, San Jose, CA, USA), with at least 100,000 lymphocyte-gated events recorded per sample. Compensation was carried out using UltraComp eBeads (Thermo Fisher Scientific, Logan, UT, USA), and downstream analyses were performed in FlowJo (Tree Star, Ashland, OR, USA).

### 2.5. Human Proteome Microarray

Proteome-wide profiling of IgG binding was conducted using HuProt™ Human Proteome Microarrays v4.0 (CDI Labs, Mayaguez, Puerto Rico). From the complete platform, a subset of 7387 proteins known to be expressed in γδT cells—identified through the Human Protein Atlas—was selected for focused analysis. These proteins are purified human sequences expressed as GST-fusion constructs in *Saccharomyces cerevisiae*. Supplementary Table S2 provides the full protein list.

Before serum incubation, microarrays were blocked for 30 min with Rockland Blocking Buffer MB-070. Serum samples (diluted 1:500) were applied and incubated overnight at 4 °C. Bound IgG was detected using DyLight-680-conjugated goat anti-human IgG (Fc-specific; 0.1 µg/mL, Thermo Fisher Scientific) for 45 min.

Slides were scanned on an InnoScan 710-IR scanner (10 µm resolution, gain 30, low laser power). Background signals, obtained from arrays probed with secondary antibody only, were subtracted from all experimental measurements. Data were processed using Mapix v9.1.0 and R v4.3.2. Foreground and background intensities were calculated, replicate spots were averaged, and protein interactions with sample-to-background ratios ≤2 were excluded. A minimum background value of 50 intensity units was applied. Specific binding was defined as signal intensities exceeding the healthy-control mean by at least three standard deviations and showing a fold increase > 2 relative to controls.

Protein interaction and pathway-enrichment analyses were carried out using STRING (Vs 12.0, 2025 release), incorporating annotations for subcellular localization (COMPARTMENTS), COVID-19-relevant pathways (Reactome), and tissue-specific expression patterns (TISSUES).

### 2.6. Statistical Analysis

Statistical analyses were performed using GraphPad Prism v8.0 (GraphPad Software, La Jolla, CA, USA). Each in vitro experiment represents an independent assay performed with PBMCs from distinct donors (10–12 experiments in total, as indicated in the figure legends). Comparisons across three or more groups were conducted using non-parametric one-way ANOVA (Kruskal–Wallis test), with statistical significance set at *p* ≤ 0.05. Differences in IgG binding intensities between groups were evaluated using the Mann–Whitney U test, with *p* < 0.05 considered significant.

## 3. Results

### 3.1. SARS-CoV-2–Induced IgG Modulates γδ T Cell Cytokine Production, Subset Distribution, and Activation Profiles

**Figure 1:**
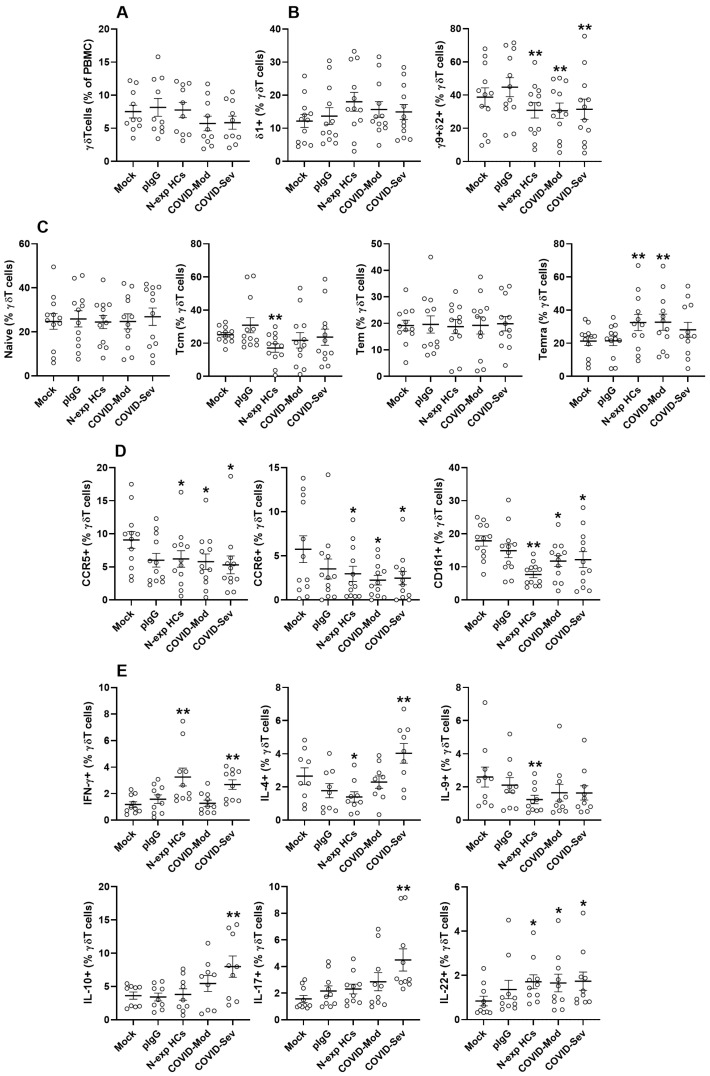
SARS-CoV-2-infected patient IgG modulates γδ T cell subset distribution, activation, and cytokine production. Peripheral blood mononuclear cells (PBMCs) from healthy donors were cultured for 3 days in RPMI 1640 supplemented with 10% fetal bovine serum under five conditions: mock (no IgG), 100 μg/mL therapeutic polyvalent IgG (pIVIg), IgG from SARS-CoV-2 non-exposed healthy controls (N-exp HCs), IgG from patients with moderate COVID-19 (COVID-Mod), or IgG from patients with severe COVID-19 (COVID-Sev). Flow cytometry was used to evaluate: (**A**) frequencies of total γδ T cells (CD3^+^γδTCR^+^); (**B**) frequencies of Vδ1^+^ and Vγ9^+^Vδ2^+^ γδ T cell subsets; (**C**) distribution of naïve (CD45RA^+^CD27^+^), central memory (Tcm; CD45RA^−^CD27^+^), effector memory (Tem; CD45RA^−^CD27^−^), and terminal effector memory (TemRA; CD45RA^+^CD27^−^) γδ T cells; (**D**) expression of CCR5, CCR6, and CD161 on γδ T cells; (**E**) intracellular production of IFN-γ, IL-4, IL-9, IL-10, IL-17, and IL-22 by γδ T cells. Statistical significance: * *p* ≤ 0.05 vs. mock; ** *p* ≤ 0.05 vs. mock and pIVIg.

To determine whether IgG from SARS-CoV-2-infected individuals influence γδ T cell maintenance, PBMCs were cultured in the presence of IgG derived from N-exp HCs, COVID-Mod, and COVID-Sev donors. Total γδ T cell frequencies and the proportion of Vδ1^+^ cells remained unchanged across all experimental conditions (Figure 1A,B). In contrast, exposure to any of the three IgG sources resulted in a significant reduction in Vγ9^+^Vδ2^+^ γδ T cells compared with mock or pIgG controls, suggesting a selective sensitivity of this subset (Figure 1B).

We next examined whether SARS-CoV-2-related IgG shapes different profiles within the γδ T cell compartment. N-exp HCs IgG significantly decreased the proportion of central memory γδ T cells while leaving naïve and Tem subsets unaltered (Figure 1C). Additionally, both N-exp HCs and COVID-Mod IgG reduced the TemRA subset relative to control conditions, which could indicate shared effects on terminally differentiated γδ T cells (Figure 1C).

Given these alterations in γδ T cell subsets, we next examined the expression of activation markers and homing-associated receptors. IgG from non-exposed healthy controls (N-exp HCs), moderate COVID-19 patients (COVID-Mod), and severe COVID-19 patients (COVID-Sev) all significantly reduced the frequencies of CCR5^+^, CCR6^+^, and CD161^+^ γδ T cells compared with mock-treated cultures. The magnitude of suppression was comparable across groups for CCR5^+^ and CCR6^+^ cells; however, IgG from N-exp HCs induced a more pronounced reduction in the CD161^+^ γδ T cell population than IgG from either COVID-Mod or COVID-Sev donors (Figure 1D).

Intracellular cytokine profiling revealed distinct immunomodulatory signatures across IgG groups. Both N-exp HCs and COVID-Sev IgG enhanced IFN-γ production, but their influence on Th2-associated cytokines diverged: N-exp HCs IgG decreased IL-4, whereas COVID-Sev IgG significantly increased its production (Figure 1E). N-exp HCs IgG uniquely suppressed IL-9 production, whereas COVID-Sev IgG selectively augmented IL-10 and IL-17 levels relative to all other conditions. Notably, all IgG sources significantly increased IL-22 production compared with the mock condition (Figure 1E).

Collectively, these findings demonstrate that SARS-CoV-2-induced IgG exerts selective and functionally diverse effects on γδ T cells. Vγ9^+^Vδ2^+^ cells appear particularly susceptible, activation and homing markers are broadly downregulated, and cytokine outputs diverge depending on the immunological background of the IgG donor. These results indicate that the IgG repertoire generated during SARS-CoV-2 infection may actively shape γδ T cell responses in a disease severity-dependent manner.

### 3.2. SARS-CoV-2-Infected Patient IgG Binds the γδ T Cell Membrane and Displays Expanded Reactivity Toward γδ T Cell-Expressed Proteins

**Figure 2:**
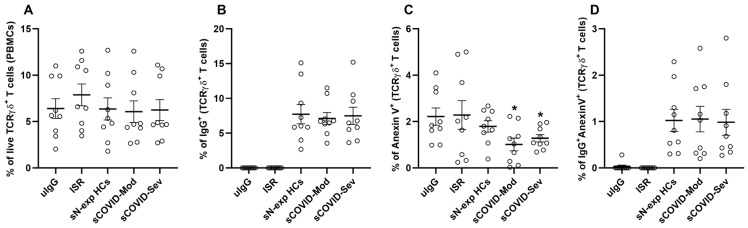
SARS-CoV-2-derived IgG displays comparable membrane-binding capacity but differential effects on early apoptosis in γδ T cells. Peripheral blood mononuclear cells (PBMCs) from healthy donors were incubated for 30 min in RPMI 1640 supplemented with 10% fetal bovine serum under five conditions: unlabeled IgG (uIgG), IgG staining reagents alone (ISR), fluorescently labeled IgG from non-exposed healthy controls (sN-exp HCs), labeled IgG from patients with moderate COVID-19 (sCOVID-Mod), or labeled IgG from patients with severe COVID-19 (sCOVID-Sev). Flow cytometry was used to quantify (**A**) the frequency of live γδ T cells, (**B**) the frequency of γδ T cells positive for membrane-bound IgG, (**C**) the frequency of γδ T cells positive for Annexin V staining, and (**D**) the frequency of γδ T cells double positive for Annexin V and membrane-bound IgG. * *p* ≤ 0.05 compared with sN-exp HCs.

To assess whether the observed functional alterations arise through direct IgG–cell interactions, we first evaluated membrane binding of labeled IgG to γδ T cells. The staining protocol itself did not affect γδ T cell frequencies (Figure 2B). Similar binding levels (5–6%) were observed for N-exp HCs, COVID-Mod, and COVID-Sev IgG (Figure 2B), indicating that differences in functional outcomes are not attributable to disparities in binding efficiency. IgG from moderate and severe COVID-19 patients (COVID-Mod and COVID-Sev) induced significantly lower frequencies of early apoptotic γδ T cells than IgG from non-exposed healthy controls (N-exp HCs; *p* < 0.05; Figure 2C). Moreover, the proportion of γδ T cells simultaneously positive for IgG and Annexin V remained at approximately 1% across all experimental conditions (Figure 2D), indicating that IgG membrane binding per se does not trigger apoptosis.

**Figure 3:**
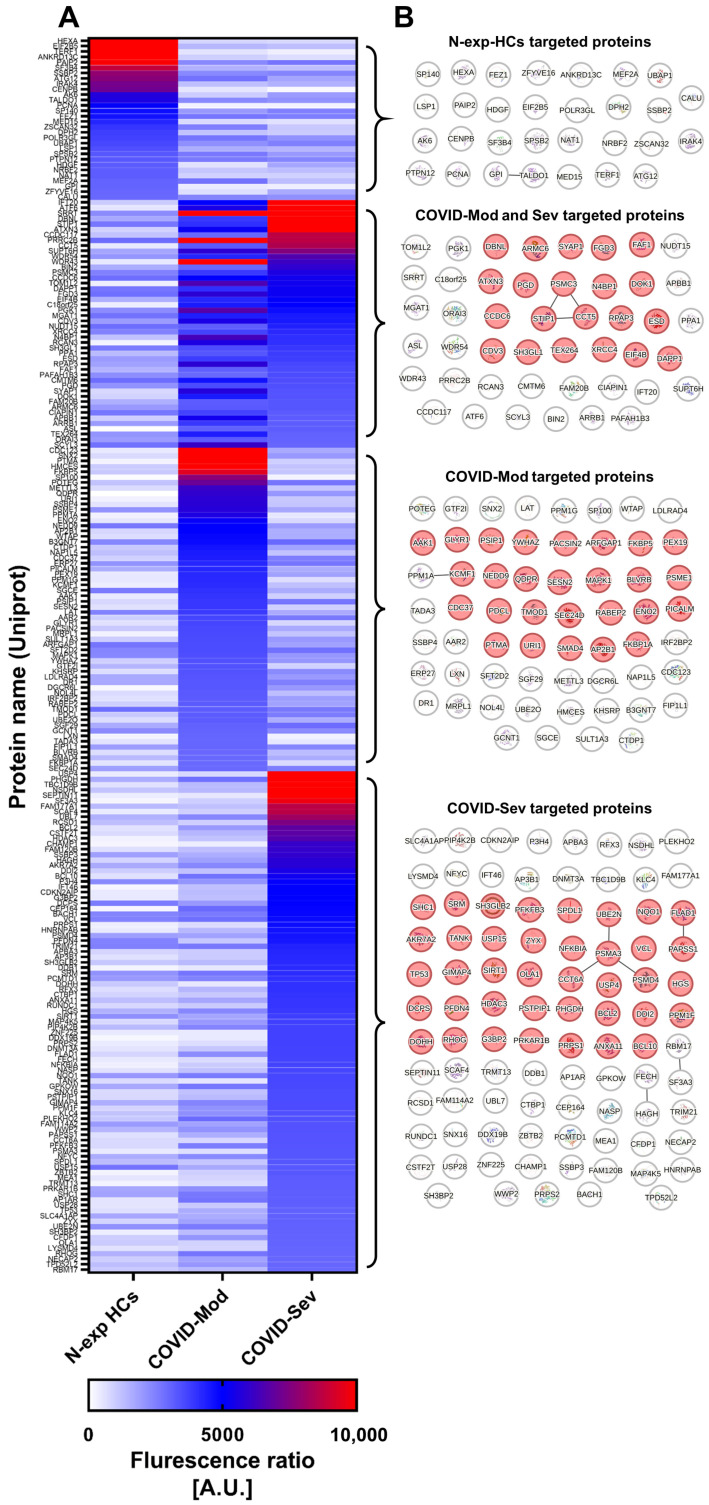
SARS-CoV-2-infected patient IgG displays expanded reactivity to γδ T cell-expressed proteins. (**A**) Heatmap summarizing γδ T cell-expressed proteins significantly recognized by at least one IgG preparation: IgG from SARS-CoV-2 non-exposed healthy controls (N-exp HCs), IgG from patients with moderate COVID-19 (COVID-Mod), or IgG from patients with severe COVID-19 (COVID-Sev). (**B**) Protein–protein interaction network (PPIN) of proteins differentially targeted by at least one of the IgG formulations. Nodes without edges represent proteins with no detectable homology. Red nodes indicate proteins enriched in the cytosolic compartment (FDR < 0.05).

We next characterized the protein targets of IgG within a panel of 7387 γδ T cell-expressed proteins. Using a stringent threshold (mean of N-exp HCs + 3 SD), N-exp HCs IgG strongly recognized 30 proteins, while a shared set of 46 proteins was targeted by both COVID-Mod and COVID-Sev IgG (Figure 3A). Disease severity further expanded idiotype diversity: COVID-Mod IgG uniquely recognized 60 proteins, whereas COVID-Sev IgG uniquely targeted 93 proteins (Figure 3A).

Sequence-homology analyses showed no detectable homology among proteins recognized exclusively by N-exp HCs IgG. In contrast, a limited number of proteins targeted by COVID-Mod or COVID-Sev IgG exhibited structural similarity, with small clusters observed in each group (Figure 3B). Subcellular localization analysis revealed significant enrichment of recognized cytosolic proteins across all SARS-CoV-2-related IgG groups.

**Figure 4:**
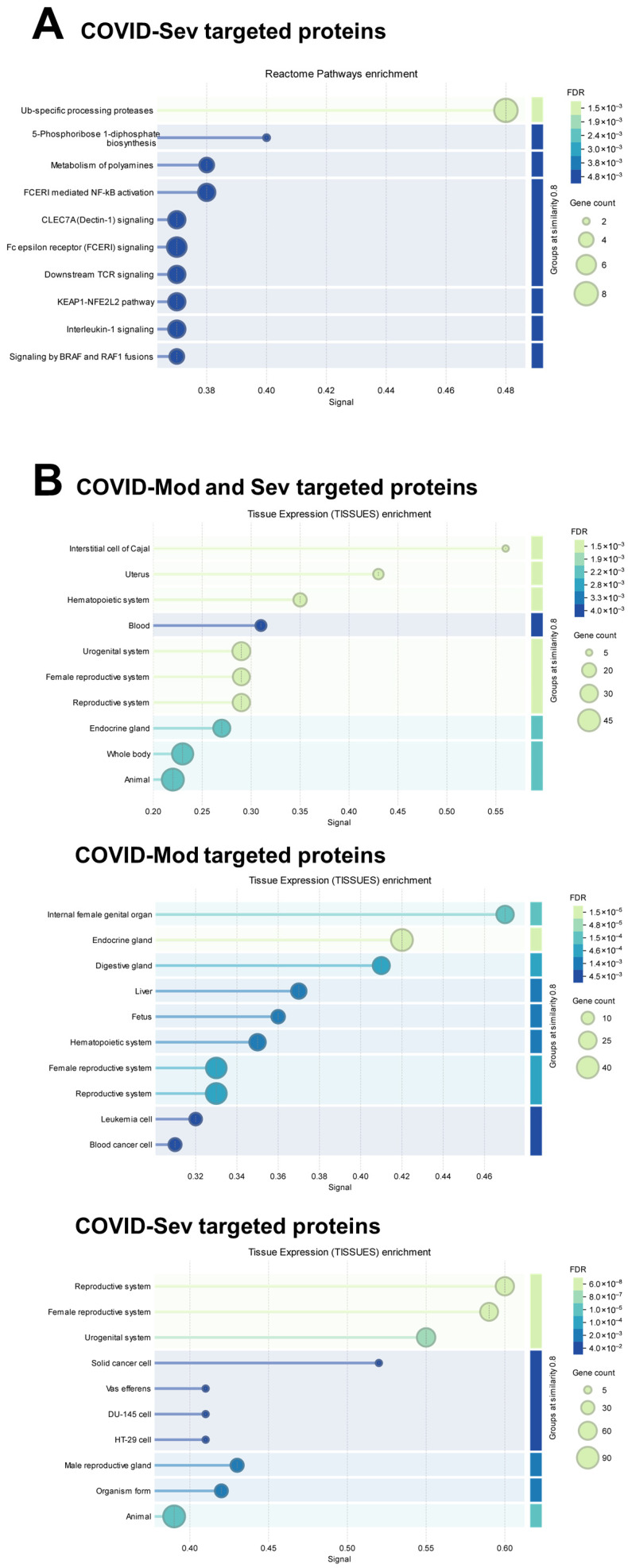
Proteins targeted by SARS-CoV-2-infected patient IgG are enriched in Reactome pathways and tissue-specific expression profiles. (**A**) Reactome pathway-enrichment analysis of IgG-targeted proteins shows statistically significant enrichment only for proteins recognized by IgG from patients with severe COVID-19 (COVID-Sev). (**B**) Tissue-enrichment analysis of IgG-targeted proteins reveals significant associations for the combined set of proteins recognized by COVID-Mod and COVID-Sev IgG, as well as for the individual COVID-Mod and COVID-Sev IgG target repertoires.

Reactome pathway mapping demonstrated that only COVID-Sev IgG showed statistically significant associations, including Ub-specific protease activity, FcεRI-mediated NF-κB activation, Dectin-1 signaling, FcεRI signaling, TCR signaling, and IL-1 signaling (Figure 4A). Tissue-enrichment analysis identified multiple significant associations with proteins expressed in diverse organs—including uterus, reproductive tissues, liver, fetal blood, urogenital tract, and endocrine glands—across the COVID-related IgG groups (Figure 4B).

Taken together, these results show that, although membrane-binding capacity is comparable across IgG groups, SARS-CoV-2 infection—particularly in severe disease—elicits an IgG repertoire with markedly expanded and distinct reactivity toward γδ T cell-expressed proteins. Only severe COVID-19 IgG displayed coherent pathway-level enrichment, suggesting stronger biological relevance and potential for functional modulation. These findings support the concept that SARS-CoV-2 infection shapes the idiotype landscape in ways that may directly influence γδ T cell biology and contribute to immune dysregulation.

## 4. Discussion

In this study, we demonstrate that IgG purified from moderate or severe COVID-19 patients exerts substantial modulatory effects on human peripheral γδ T cells. SARS-CoV-2-induced IgG selectively reduced the frequency of circulating Vγ9^+^Vδ2^+^ cells, altered memory differentiation by decreasing Tcm and TemRA subsets, downregulated key homing and activation markers (CCR5, CCR6, CD161), and shaped cytokine production—including IFN-γ, IL-4, IL-9, IL-10, IL-17, and IL-22. These functional alterations were due to direct IgG binding to γδ T cells and by a broadened spectrum of γδ T cell-associated protein targets, particularly in moderate and severe COVID-19 IgG groups. Collectively, these findings reinforce the concept that SARS-CoV-2 infection gives rise to IgG idiotypes with active immunoregulatory capacity, expanding their functional relevance beyond classical antiviral roles.

Our results align with and extend prior evidence that polyclonal IgG repertoires can modulate lymphocyte behavior through antigen-independent, idiotype-driven mechanisms. Functional studies have shown that IgG from COVID-19 patients alters MAIT-cell frequency and cytokine production [37]. Likewise, the “hooks without bait” theory posits that naturally occurring IgG networks continuously influence T and B cell maturation and function [36], and subsequent theoretical works proposed IgG as a potential natural ligand for the γδTCR, capable of shaping γδ T cell immunoregulatory circuits [39]. High-resolution proteomic and epitope-profiling studies further support this immunomodulatory framework by demonstrating that SARS-CoV-2 infection and vaccination generate broad idiotype networks directed against intracellular proteins and diverse pathogen-derived epitopes [12,13,14].

Within this evolving paradigm, our study identifies γδ T cells as functionally responsive targets of SARS-CoV-2-induced IgG, providing a direct link between idiotype network composition and γδ T cell behavior. Several lines of independent evidence highlight the importance of γδ T cells in COVID-19.

Natural killer (NK) and natural killer T (NKT) cells are well-established components of the innate and innate-like immune response to SARS-CoV-2 and have been more extensively characterized than γδ T cells in COVID-19. Multiple studies have consistently reported numerical depletion, functional exhaustion, and phenotypic remodeling of circulating NK cells in moderate and severe disease, often associated with impaired cytotoxicity, altered interferon signaling, and disease severity [40,41]. NKT cells similarly display activation-associated phenotypic changes and reduced frequencies in peripheral blood, reflecting their recruitment to inflamed tissues and their participation in early antiviral and immunoregulatory responses [40]. Importantly, recent work highlights that γδ T cells share substantial functional and transcriptional overlap with NK and NKT cells, positioning them within a coordinated network of cytotoxic and cytokine-producing lymphocytes that bridges innate and adaptive immunity [42]. Importantly, however, γδ T cells are distinguished by their strong tissue-adaptive capacity, including the acquisition of resident-like phenotypes and specialized functions in epithelial surveillance, barrier maintenance, and tissue repair, as demonstrated by fate-mapping and transcriptional studies [33]. In this context, the comparatively limited and sometimes inconsistent characterization of γδ T cells in COVID-19 likely reflects both their rapid redistribution to tissues and their context-dependent functional states, rather than a lack of biological relevance. Our findings therefore complement existing NK and NKT literature by identifying γδ T cells as antibody-responsive effectors within the same innate-like immune axis, revealing an additional layer of regulation mediated by SARS-CoV-2-induced IgG repertoires.

Importantly, the molecular and proteomic patterns identified in our array analyses should be interpreted in the context of shared immune programs across innate-like lymphocyte populations. Several of the pathways and intracellular targets recognized by SARS-CoV-2-induced IgG have been previously associated with iNKT cell activation, dysfunction, and immunopathology in COVID-19, a cell population that has been more extensively studied in this setting. Our data do not suggest that γδ T cells act in isolation or replace iNKT cells as principal mediators of pathology. Rather, the molecular array supports the existence of convergent regulatory circuits affecting multiple innate-like lymphocytes, including iNKT, NK, and γδ T cells. In this framework, γδ T cells emerge as antibody-responsive effectors that share core inflammatory and cytotoxic programs with iNKT cells but display distinct tissue-adaptive and functional plasticity. Thus, while iNKT cells have been more prominently linked to COVID-19 pathogenesis in prior studies, our findings expand this model by identifying γδ T cells as additional, IgG-sensitive components of the same immunopathological axis.

COVID-19 patients frequently present with reduced circulating Vδ2^+^ γδ T cells and an activated, pro-inflammatory phenotype that can be modulated pharmacologically, such as by statin treatment [43]. Transcriptional studies across viral diseases have identified distinct γδ T cell states and rewired effector/regulatory programs associated with infection [44]. In nonhuman primates, early expansion and activation of Vδ1^+^ γδ T cells correlate with viral replication and airway inflammation [45]. In humans, activation of NK cells and unconventional T cells—including γδ T cells—has been documented in pregnant women infected with SARS-CoV-2 [42]. Moreover, macrophage-focused studies demonstrate that SARS-CoV-2-infected macrophages can alter γδ Tcell responses, underscoring the interconnected nature of innate and unconventional T cell immunity [46].

Against this backdrop, our observation that COVID-19 IgG downregulates γδ cell homing markers and shifts cytokine secretion profiles suggests an additional antibody-mediated layer of γδ T cell regulation. The elevation of IFN-γ and IL-22, alongside the selective modulation of IL-4, IL-9, IL-10, and IL-17, reflects the capacity of distinct IgG repertoires to instruct γδ T cell effector functions. These findings are consistent with single-cell analyses showing transcriptionally diverse γδ T cell states in COVID-19 [44] and with clinical evidence that their phenotype is pharmacologically adjustable [43]. Our data therefore support a model in which idiotype networks directly contribute to the plasticity of γδ T cell responses during SARS-CoV-2 infection.

Vaccination and heterologous viral exposures further illustrate the dynamic interplay between γδ T cells and COVID-19-related immunity. Longitudinal single-cell studies of mRNA COVID-19 vaccination revealed selective expansion of memory-like Vδ2 T cells and induction of AP-1–driven transcriptional programs following booster immunization [47]. Population-based analyses show that low-baseline γδ T cell numbers are associated with increased susceptibility to SARS-CoV-2 infection despite vaccination [48]. Additionally, experimental evidence suggests that respiratory syncytial virus infection may confer heterologous protection against SARS-CoV-2 through γδ T cell-mediated trained immunity [49]. Our findings provide a mechanistic complement to these observations by showing how infection-induced IgG repertoires can directly shape γδ T cell differentiation and effector function, potentially influencing vaccine responsiveness and breakthrough infections.

The mechanisms governing γδ T cell engagement during SARS-CoV-2 infection remain incompletely understood. Studies using recombinant spike and nucleocapsid proteins suggest that viral antigens alone do not directly activate human γδ T cells, implicating secondary inflammatory pathways or complex antigenic contexts in γδ T cell activation [50]. Here, we demonstrate that IgG from COVID-19 patients binds directly to the γδ T cell membrane and recognizes a broad range of intracellular γδ T cell proteins. The proteomic patterns observed—particularly the more complex repertoire in the severe COVID-19 group—mirror independent findings of extensive autoreactive IgG responses induced by infection or vaccination [12,13]. Severity-dependent epitope-profiling studies similarly show that SARS-CoV-2 infection shapes IgG reactivity toward both viral and unrelated pathogen epitopes [14]. Collectively, these data suggest that γδ T cells serve as functional nodes within the broader IgG idiotype network elicited by SARS-CoV-2.

From a conceptual standpoint, these findings support the idea that IgG operates as a context-dependent “language” of immune regulation, encoding donor-specific immunological information that can be interpreted by lymphocytes [36,39]. The distinct functional and proteomic outcomes induced by N-exp HC, COVID-Mod, and COVID-Sev IgG likely reflect differences in idiotype composition, thereby providing γδ T cells with varying regulatory instructions. Multi-omics and proteomic analyses of SARS-CoV-2-induced IgG networks reinforce this view by revealing system-wide idiotype responses enriched for pathways related to antiviral defense, inflammation, and tissue injury [12,13]. Our findings extend these insights by demonstrating that such idiotype-encoded information can be functionally translated into altered γδ T cell subsets, its activation states, and cytokine production.

In addition to idiotype- and paratope-dependent interactions, IgG Fc glycosylation represents another important layer of antibody-mediated immune regulation. Alterations in Fc glycosylation have been reported in inflammatory and infectious diseases, including COVID-19 [51,52], and can influence antibody effector functions, receptor engagement, and downstream immune responses [53]. Although the present study focused on the functional consequences of bulk IgG idiotype diversity and protein target recognition, differences in glycosylation patterns across IgG pools may also contribute to the observed immunomodulatory effects on γδ T cells. As Fc glycosylation was not directly assessed here, its specific contribution cannot be resolved from our data and warrants investigation in future studies.

## 5. Study Limitations

Several limitations warrant consideration. Our experiments were performed in vitro, which may not fully replicate the complex in vivo milieu of acute or convalescent COVID-19. Although we identified IgG binding to γδ T cell membranes and numerous intracellular protein targets, the precise receptors mediating these interactions remain unknown. Determining whether γδTCRs, Fc receptors, or other surface molecules participate in IgG recognition is an important next step, particularly in light of theoretical models proposing IgG as a γδTCR ligand [39]. Furthermore, clinical correlations linking IgG-driven γδ T cell modulation to disease outcomes, vaccine responses, or post-COVID conditions remain to be established. Longitudinal and mechanistic studies integrating phenotyping, vaccination status, and outcome data will be essential for translation [43,47,48]. In addition, while therapeutic IVIG was included as a reference control in key functional assays, it was not applied across all experimental readouts, reflecting the study’s focus on severity-dependent differences among COVID-19-derived IgG pools.

## 6. Conclusions

Despite these caveats, our findings suggest that SARS-CoV-2-induced IgG repertoires are active participants in shaping γδ T cell immunity. By linking idiotype network architecture to γδ T cell function, this work contributes to a broader understanding of how humoral immunity can modulate unconventional T cell responses—particularly γδ T cells—during COVID-19 through IgG-dependent mechanisms. Moreover, these observations raise the possibility that systematic characterization of IgG idiotype repertoires, rather than focusing exclusively on individual antigens, may provide new insights into antibody-mediated immune regulation and inform future immunomodulatory strategies in COVID-19 and related conditions.

## Data Availability

The data that support the findings of this study are available from the corresponding author, JRV, upon reasonable request.

## Supplementary Materials

The following supporting information can be downloaded at: https://www.mdpi.com/article/10.3390/cells15050401/s1, Table S1: Information about the patients with COVID-19; Table S2: Comprehensive list of proteins expressed in γδ T cells, as identified from the Human Protein Atlas, that were evaluated for IgG reactivity in this study.
